# Retraction Note: Efficacy of a multidimensional self-management intervention on low-education women with metabolic syndrome: a cluster randomized controlled trial

**DOI:** 10.1038/s41598-024-82509-1

**Published:** 2024-12-10

**Authors:** Shu-Hung Chang, Yi-Ya Chang, Wen-Juei Jeng, Jackson Pui Man Wai

**Affiliations:** 1 Graduate Institute of Gerontology and Health Care Management, Geriatric and Long-Term Care Research Center, Chang Gung University of Science and Technology, Taoyuan, Taiwan; 2 Department of Gastroenterology and Hepatology, Chang Gung Medical Foundation, Linkou Chang Gung Memorial Hospital, Taoyuan, Taiwan; 3 Department of Nursing, Chang Gung University of Science and Technology, Taoyuan, Taiwan; 4 Department of Health Management, Chang Gung Medical Foundation, Taipei Chang Gung Memorial Hospital, Taipei, Taiwan; 5 College of Medicine, Chang Gung University, Taoyuan, Taiwan; 6 Department of Nutrition Science, Fu Jen Catholic University, New Taipei City, Taiwan; 7 Graduate Institute of Sport Science, National Taiwan Sport University, Taoyuan, Taiwan

Retraction of: *Scientific Reports* 10.1038/s41598-023-36971-y, published online 26 June 2023

The Editors have retracted this Article.

After publication of this study concerns were raised by the readers who were unable to reproduce the results using the methods described in the Article. The Authors appear to have also provided the readers with an incomplete dataset that did not contain data on medication use by the patients. Following post-publication peer review by the journal, the Authors did not respond to requests for clarifications or to provide the missing data. As a result, the Editors no longer have confidence in the findings reported in the Article.

None of the Authors have responded to correspondence from the Editors about this retraction.

